# Exogenous Calcium Suppresses the Oviposition Choices of *Frankliniella occidentalis* (Thysanoptera: Thripidae) and Promotes the Attraction of *Orius similis* (Hemiptera: Anthocoridae) by Altering Volatile Blend Emissions in Kidney Bean Plants

**DOI:** 10.3390/insects13121127

**Published:** 2022-12-06

**Authors:** Wan-Qing Huang, Guang Zeng, Jun-Rui Zhi, Xin-Yue Qiu, Zhen-Juan Yin

**Affiliations:** 1 Guizhou Provincial Key Laboratory for Agricultural Pest Management in the Mountainous Region, Institute of Entomology, Guizhou University, Guiyang 550025, China; 2 Department of Resources and Environment, Moutai Institute, Renhuai 564507, China

**Keywords:** *Frankliniella occidentalis*, *Orius similis*, exogenous calcium, induced defense, herbivore-induced plant volatiles, olfactory response, gas chromatography–mass spectrometry

## Abstract

**Simple Summary:**

The western flower thrips, *Frankliniella occidentalis* (Pergande) (Thysanoptera: Thripidae) is an economically damaging invasive pest that is difficult to control due to their resistance to insecticides. Utilizing plant-induced defense responses is a new strategy to increase plant resistance to *F. occidentalis*. A growing number of studies have shown that calcium plays an important role in enhancing plant resistance to pest-induced stresses. In this study, we found that exogenous calcium treatment could alter the composition of volatile compounds in kidney bean plants, repelling *F. occidentalis* while attracting its natural enemy, *Orius similis* Zheng (Hemiptera: Anthocoridae), and reducing the oviposition preference choice of *F. occidentalis*. This study provides a theoretical basis for the control of *F. occidentalis*.

**Abstract:**

*Frankliniella occidentalis* is a destructive pest of horticultural plants, while *Orius similis* is a natural enemy of thrips. It has been demonstrated that exogenous calcium could induce plant defenses against herbivore attack. We examined whether CaCl_2_ supplementation altered the volatile emissions of kidney bean plants, which influence the oviposition preference of *F. occidentalis*. We also assessed the influence of volatile cues on *O. similis*. Using Y-tube olfactometer tests, we found that exogenous CaCl_2_ treatment inhibited the selectivity of *F. occidentalis* but attracted *O. similis*. In addition, CaCl_2_ treatment reduced the oviposition preference of *F. occidentalis*. Gas chromatography–mass spectrometry analyses revealed that CaCl_2_ treatment altered the number and relative abundance of the volatile compounds in kidney bean plants and that (E)-2-hexen-1-ol, 1-octen-3-ol, β-lonone, and (E,E)-2,4-hexadienal might be potential olfactory cues. Furthermore, the results of the six-arm olfactometer test indicated that 1-octen-3-ol (10^−2^ μL/μL), β-lonone (10^−2^ μL/μL), and (E,E)-2,4-hexadienal (10^−3^ μL/μL) repelled *F. occidentalis* but attracted *O. similis*. Overall, our results suggested that exogenous CaCl_2_ treatment induced defense responses in kidney bean plants, suggesting that CaCl_2_ supplementation may be a promising strategy to enhance the biological control of *F. occidentalis*.

## 1. Introduction

Calcium is an essential nutrient for plant growth and development, as well as a secondary messenger in plant cells [1,2,3]. Accumulating evidence has shown that calcium plays an important role in improving plant resistance to abiotic and biotic stresses [4,5]. Supplementation with the appropriate concentrations of exogenous calcium improves plant resistance to abiotic stresses, including salt stress, drought, extreme temperatures, and heavy metals [6,7,8,9,10,11]. In addition, exogenously supplemented calcium can regulate the resistance of plants to biotic stresses. Calcium supplementation can induce different chemical reactions in plants to activate defense enzymes, such as lipoxygenase, polyphenol oxidase, and phenylamine ammonia lyase, thereby enhancing plant resistance to pests [12,13]. In poinsettia, calcium treatment significantly reduces the survival rate of *Bemisia tabaci* (Gennadius) (Hemiptera: Aleyrodidae) and prolongs its development time, thus reducing damage to the plants [14]. The treatment of rice plants with exogenous calcium enhances their resistance to *Nilaparvata lugens* (Stål) (Hemiptera: Delphacidae) and reduces its survival rate [13]. Similarly, the treatment of wheat plants with exogenous calcium prolongs the development time of aphids and reduces their feeding rate [11].

Plants defend themselves against herbivorous insects via various constitutive and induced defense mechanisms, and these defense mechanisms are mediated mainly by external stimuli or exogenous substances [15,16]. Responses to external stimuli or exogenous substances include the accumulation of defensive compounds and the release of volatile organic compounds (VOCs). VOCs play an important role as signals in plant defense to regulate the behavioral responses of herbivores and natural enemies [17,18,19].

Growing evidence has indicated that exogenous biotic and abiotic elicitors trigger plant defensive responses and alter the composition of VOCs to increase the repulsion of herbivores and the attraction of their predators [20,21,22,23,24,25]. For example, when plants are attacked by herbivorous insects, they release various volatile compounds, which are known as herbivore-induced plant volatiles (HIPVs) [26,27]. HIPVs not only poison or repel herbivorous insects in direct defense but also provide plants with indirect defense by attracting natural enemies of the pests. Therefore, HIPVs are considered signal substances of tritrophic interaction [22,28,29,30]. Several studies have also reported that abiotic elicitors, such as copper, (Z)-3-hexenol, and silicon, can trigger plants to produce larger quantities of induced volatile compounds [24,25,31,32].

*Frankliniella occidentalis* (Pergande) (Thysanoptera: Thripidae) is a native species in the western part of North America [33]. As an economically damaging invasive pest, *F. occidentalis* not only causes direct damage by feeding and laying eggs in plants but also spreads a variety of plant viruses, thereby posing a serious threat to vegetables and ornamental flowers [34]. *F. occidentalis* has spread to several regions in mainland China [35], and the pest has proven difficult to control. Although *F. occidentalis* is controlled using insecticides, its resistance to insecticides is a long-term threat [36,37]. Hence, a combination of more diverse strategies is needed to manage *F. occidentalis* [38,39]. Utilizing plant-induced defenses against *F. occidentalis* may represent an alternative approach for crop protection, while being environmentally and natural enemy friendly [37].

*Orius* (Hemiptera: Anthocoridae) is a genus of omnivorous predators that prey on the eggs of various lepidoptera and small insect pests, such as thrips, aphids, and mites [40,41,42]. Several *Orius* species are commercially produced as biocontrol agents to protect various crops worldwide [41,43]. In China, *Orius similis* Zheng is widely distributed as a dominant species in southern farmlands [44,45]. It is mainly used for controlling thrips due to its high fertility, high predation and wide predation range, and proven efficacy as a biological control agent of *F. occidentalis* [45,46,47]. Studies have shown that *O. similis* locates its prey by means of olfactory cues emitted by prey-infested plants [42,48].

Previous studies have shown that exogenous calcium supplementation significantly increases defense enzyme activity in kidney bean plants, as well as reducing the choice of *F. occidentalis* [12,49]. However, the effect of calcium treatment on the production of volatiles in kidney bean plants, and the potential effect on the indirect defense of *F. occidentalis*, remain unclear. We hypothesized that calcium-treated kidney bean plants have altered volatile proportional compositions, which enhance the repulsion of *F. occidentalis* and the attraction of natural enemies. Therefore, in this study, our objectives are to investigate the impacts of calcium supplementation on: (i) the behavioral responses of *F. occidentalis* and its natural enemy, *O. similis*, and the oviposition preference of *F. occidentalis*; (ii) potential alterations in plant volatile emissions; and (iii) the responses of thrips and *O. similis* to identified plant volatile compounds. The outcomes of this study will further clarify the role of calcium as an inducing factor in the control of *F. occidentalis* and may help in the development of effective environmentally friendly strategies for sustainable management of *F. occidentalis*.

## 2. Materials and Methods

### 2.1. Plants

Healthy seeds of kidney bean plants (*Phaseolus vulgaris* L.) ‘Daoxiaomei’ were obtained from the Shenyang Xinjia Food Sales Co., LtD. Liaoning Province, China. Seeds sterilized with 10% H_2_O_2_ for 15 min and then rinsed with sterile distilled water. After a day of presoaking, seeds were transferred into plastic pot (Model: WGLL-125BE, Taisite Instrument Co., Ltd. Tianjin, China) (1 seed per planter; diameter, 12 cm; height, 15 cm) with nutrient soil, which has been autoclaved at 150 °C. All plants were placed in an environment chamber at 25 °C for 14 h using a 10-h light/dull photoperiod and 75% ± 5% relative humidity (RH). In the above conditions, plants were watered every day in order to maintain soil moisture. No pesticide was applied during the growth period. Plants with three leaves at day 22 were used for the following trials.

### 2.2. Test Insects

*F. occidentalis* populations were collected from kidney bean plants at the experimental farm of Guizhou University in Guiyang, China, in June 2018. Shaking the flowers and upper leaves of the kidney bean plant to make the thrips fall into the insect box (20.6 × 13.4 × 9.3 cm) with kidney bean pods. Firstly, preliminarily determined the thrips species according to morphology and body color by the naked eye, then these thrips were kept to rear for one generation, and other thrips were killed. Then, 30 female adults were randomly chosen among these thrips for slide specimen according to the method of Zhang et al. [50] by maceration, washing, dehydration, mounting, and desiccation. The thrips was identified under the microscope according to the methods of Wang et al. [51]. After accurate identifying the thrips was *F. occidentalis*, the western flower thrips were continuously reared in the laboratory for more than 50 generations. Slide specimens were deposited in the Institute of Entomology, Guizhou University. Thrips were kept in a climate chamber with kidney bean pods as the food source under the same conditions described above.

*O. similis* populations were obtained from vegetable fields at the experimental farm of Guizhou University in Guiyang, China. The species was identified according to the external genitals by the method of Zhang et al. [52]. They were raised in a plastic case (30 cm long, 23 cm wide, and 18 cm high) and a climate chamber under the conditions as same as above. *O. similis* populations were provided with adequate numbers of *F. occidentalis* 2nd instar larvae and *Sitotroga cerealella* (Olivier) (Lepidoptera: Gelechiidae) eggs in a ratio of 1 to 1, and food was changed daily. *S. cerealella* eggs were purchased from the Shandong Academy of Agricultural Sciences, Shandong, China.

### 2.3. Plant Treatment Settings

Healthy and consistent kidney bean plants were selected for the following treatments. Four different treatments were applied to plants: CaCl_2_, H_2_O (control), Western flower thrips (WFT)+CaCl_2_, and WTF+H_2_O. In the CaCl_2_ group, 15 mL of 20 mM CaCl_2_ solution were sprayed on an entire kidney bean plant (labeled CaCl_2_ treatment). Control plants were sprayed with the same volume of distilled water (labeled H_2_O treatment). Twenty larvae of second-instar *F. occidentalis* were inoculated on the leaves of kidney bean plants in the WFT group’s 1 day after being sprayed with either H_2_O or CaCl_2_. After thrips had infested for 24 h, the treated kidney bean plants (labeled H_2_O+WFT and CaCl_2_+WFT) were used for the following experiments. All treatments were performed in a climatic chamber under the conditions described above.

### 2.4. Y-Tube Olfactometer Bioassays

The olfactory responses of thrips were tested using a Y-tube olfactometer as described by Cao et al. [53]. 2–3 days old adult female thrips were starved for 4 h before the application of the treatments. A total of 10 treatment combinations were compared: (1) clean air versus H_2_O; (2) clean air versus H_2_O+WFT; (3) clean air versus CaCl_2_; (4) clean air versus CaCl_2_+WFT; (5) H_2_O versus H_2_O+WFT; (6) H_2_O versus CaCl_2_; (7) H_2_O versus CaCl_2_+WFT; (8) WFT versus CaCl_2_; (9) H_2_O+WFT versus CaCl_2_+WFT; (10) CaCl_2_ versus CaCl_2_+WFT. For each comparison, 100 female thrips were tested. The flow rate was set at 300 mL/min. All bioassays were performed between 9:00 am and 6:00 pm under the following conditions: 25 °C ± 2 °C, 75 °C ± 5% RH, and 1000 lux illumination. Kidney bean plants were replaced for every 10 individuals tested. During the test, a 15-W fluorescent lamp was placed directly above the Y-olfactory instrument for balanced illumination to reduce the influence of natural light changes on the tropism test.

The olfactory response test was repeated with the same parameters for 2–3 days old female adults *O. similis*, except that for each comparison, 60 females were tested.

### 2.5. Free Choice Test for Oviposition Preference of F. occidentalis

In this experiment, a plant treated with calcium and a plant treated with water (the treatment was same with 2.3) were placed in a square cage (200 mesh, 50 × 50 × 50 cm), with a spacing of 30 cm between the centers of two pots. The soil surfaces were covered with aluminum foil to avoid the influence of volatile compounds on the selection of *F. occidentalis*. A total of 50 adult female thrips (5 days old) were put inside a tube, then the tube was placed on the ground halfway between the H_2_O-treated pot and the calcium-treated pot. Thrips were allowed to choose between the two plants to lay their eggs. After 48 h, the adult thrips were removed, and each plant was transferred to a clean insect-free transparent bucket. The number of hatched larvae on the plants was observed and recorded every 12 h under dissecting microscope until no larvae were present. Finally, the number of hatched larvae on each treated plant was counted and estimated as the number of eggs laid [54]. The experiment was conducted in a climate chamber using the conditions described above. The test was replicated 10 times at the same time.

### 2.6. Analysis of Plant Volatiles

Kidney bean plant volatiles from the H_2_O, H_2_O+WFT, CaCl_2_, and CaCl_2_+WFT plants were collected and analyzed as described by Cao et al. [53]. One kidney bean leaf (2.5 g) excised from the plant was put into a triangular glass bottle (25 mL), and the mouth was sealed with a parafilm membrane. The manual sample injector of the solid phase microextraction instrument fiber head was inserted into the bottle (2 cm, ~50/30 μm DVB/CAR/PDMS StableFlex). After headspace extraction at 80 °C for 60 min, remove the extraction head and immediately insert it into the sample inlet of the gas chromatograph (at 250 °C), and then thermally analyze the sample. The chromatographic column was HP-5 MS (60 m × 0.25 mm × 0.25 μm) elastic Shi Ying capillary column, the initial temperature was 40 °C (retention time was 2 min), the temperature was raised to 180 °C at 10 °C/min, and then to 310 °C at 10 °C/min, and the running time was 55 min. The mass spectra of compounds were compared with those in databases (Nist 20 and Wiley 275), and the relative mass fraction of each chemical component was determined using the peak area normalization method. The test was replicated three biological replicates per one treatment, and one bean plant for one replicate.

### 2.7. Volatiles Identified with Test Chemicals

The changes in compounds may affect the behavioral responses of the thrips and predatory bug. By gas chromatography–mass spectrometry (GC–MS) analysis, the compounds had significant alterations in the proportional composition among different treatments would be chosen for further test. The effect of different concentrations of chosen compounds on thrips and predatory bug would be tested using a six-armed olfactometer bioassay. 

Mineral oil (Aladdin, Shanghai, China) solutions of (E)-2-hexen-1-ol, β-lonone (Aladdin, Shanghai, China; chemical purity 97%), 1-octen-3-ol (Aladdin, Shanghai, China; chemical purity 98%), and (E,E)-2,4-hexadienal (Aladdin, Shanghai, China; chemical purity 95%) were prepared (10^−5^, 10^−4^, 10^−3^, 10^−2^, and 10^−1^ μL/μL) [55]. Solutions were stored at 4 °C until testing.

### 2.8. Olfactory Assay with Synthetic Standards 

Following the method described by Cao et al. [55], a six-arm olfactometer was used to evaluate the behavioral responses of adult *F. occidentalis* to different doses of (E)-2-hexen-1-ol, 1-octen-3-ol, β-lonone, and (E,E)-2,4-hexadienal respectively. The olfactometer was consisted of a central chamber with six arms, each of which was connected to a glass tube that extended forth at an equal distance and at an identical angle (60°). Each arm was attached to a pear-shaped glass bottle carrying an odor source or control through Teflon tubing. In each experiment, 25 uL was used for each of 5 chemical solutions and, mineral oil was used as the control. The solutions were absorbed onto a disk of filter paper with a diameter of 2.0 cm to serve as the odor source and control, respectively. To direct the odor source to thrips, set the airflow of each arm to 200 mL/min. Prior to the experiment, *F. occidentalis* (2–3 day old females) were starved for 4 h, partitioned into gatherings (200 individuals per group), and imported into the central chamber using a funnel. Insects that entered one arm of the olfactometer within 20 min were considered to have pursued a specific scent, while thrips that entered no arm within this time were considered non-choice. Five replications were performed for each treatment. The arms were cleaned with 75% ethanol and dried and were rotated (60°) after each test. The six-arm bioassay was conducted from 10:00 a.m. to 18:00 p.m. The chamber was illuminated with a 15 W fluorescent lamp which was 40 cm above central chamber.

The olfactory response test was repeated in the same manner for *O. similis*, except that for each comparison, 40 females were tested per group.

### 2.9. Statistical Analyses

All statistical analyses were performed using the SPSS version 22.0 (SPSS Inc., Chicago, IL, USA). Y-tube olfactory response and oviposition choice test data were analyzed using a *χ*^2^ goodness-of-fit test (** *p* < 0.01, * *p* < 0.05). Plant volatile analysis and six-arm olfactometer test data were analyzed using one-way ANOVA and Tukey’s honestly significant difference test (*p* < 0.05).

## 3. Results

### 3.1. Y-Tube Olfactometer Bioassays

**Figure 1:**
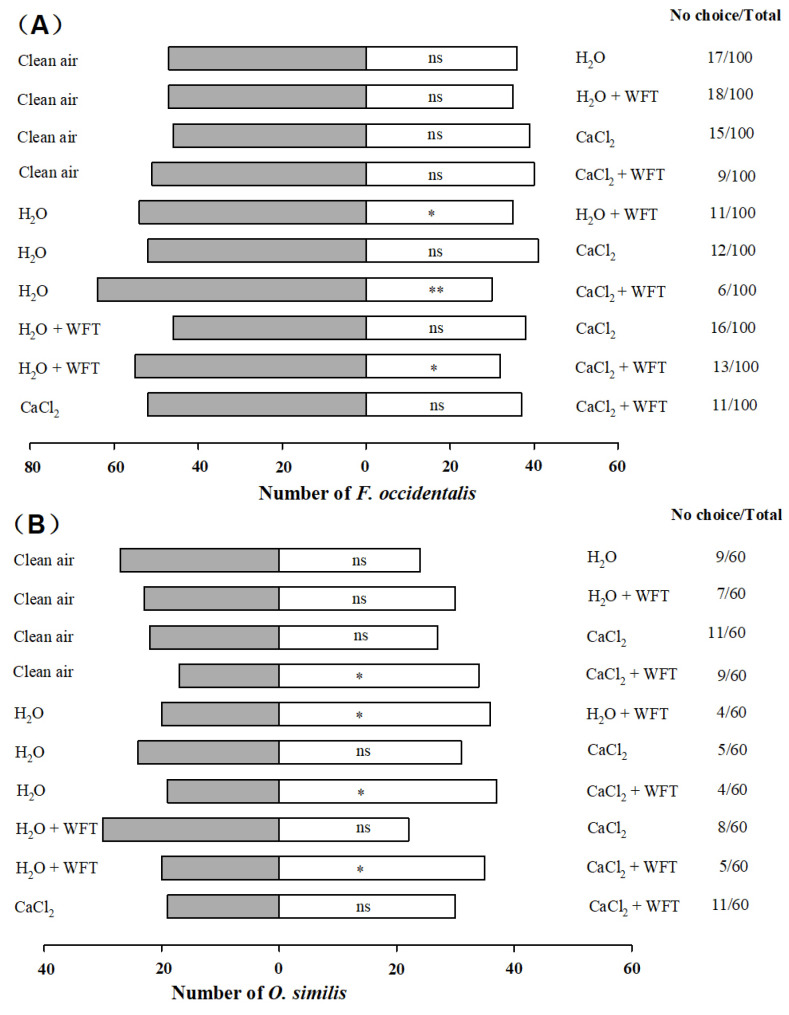
Olfactory responses of *F. occidentalis* (**A**) and *O. similis* (**B**) to volatiles from five test sources. Asterisks indicate significant differences based on *χ*^2^ test (** *p* < 0.01, * *p* < 0.05). “ns” represents no significant difference (*p* > 0.05).

The results of the Y-tube olfactometer bioassay showed that *F. occidentalis* had significantly different responses to the different plant treatments (Figure 1). When *F. occidentalis* adults were presented with the choice of kidney bean plants or clean air, thrips showed no significant preferences for H_2_O (*χ*^2^ = 1.458, *df* = 1, *p* = 0.227), H_2_O+WFT (*χ*^2^ = 1.756, *df* = 1, *p* = 0.185), CaCl_2_ (*χ*^2^ = 1.301, *df* = 1, *p* = 0.254), or CaCl_2_+WFT (*χ*^2^ = 1.330, *df* = 1, *p* = 0.249) and clean air. *F. occidentalis* significantly preferred volatiles from H_2_O compared with H_2_O+WFT (*χ*^2^ = 4.056, *df* = 1, *p* = 0.044) and CaCl_2_+WFT (*χ*^2^ = 12.298, *df* = 1, *p* < 0.01), but there was no significant difference between H_2_O and CaCl_2_ (*χ*^2^ = 1.301, *df* = 1, *p* = 0.254). When *F. occidentalis* was presented with choices among different treatments, thrips showed significantly lower preferences for CaCl_2_+WFT than for H_2_O+WFT (*χ*^2^ = 6.080, *df* = 1, *p* = 0.014). Thrips showed no significant preferences in the other pairings of the different treatments, such as H_2_O+WFT versus CaCl_2_ (*χ*^2^ = 0.762, *df* = 1, *p* = 0.383) and CaCl_2_ versus CaCl_2_+WFT (*χ*^2^ = 2.528, *df* = 1, *p* = 0.112).

*O. similis* adults showed significantly different responses to different plant treatments (Figure 1). When *O. similis* adults were presented with the choice of different treatments of kidney bean plants or clean air, they showed a significant preference only to the CaCl_2_+WFT-treated plants (*χ*^2^ = 5.667, *df* = 1, *p* = 0.017). There was no significant difference between clear air with H_2_O (*χ*^2^ = 0.176, *df* = 1, *p* = 0.674), H_2_O+WFT (*χ*^2^ = 0.925, *df* = 1, *p* = 0.336), and CaCl_2_ (*χ*^2^ = 0.510, *df* = 1, *p* = 0.475). When *O. similis* was presented with choices among different treatments, they showed significant preferences for H_2_O+WFT (*χ*^2^ = 4.571, *df* = 1, *p* = 0.033) and CaCl_2_+WFT (*χ*^2^ = 5.786, *df* = 1, *p* = 0.016) compared with H_2_O alone. CaCl_2_+WFT was more attractive to *O. similis* than H_2_O+WFT (*χ*^2^ = 4.091, *df* = 1, *p* = 0.043). There was no significant different between the H_2_O and CaCl_2_ treatments (*χ*^2^ = 0.891, *df* = 1, *p* = 0.345). In the other pairings, such as H_2_O+WFT versus CaCl_2_ (*χ*^2^ = 1.231, *df* = 1, *p* = 0.267) and CaCl_2_ versus CaCl_2_+WFT (*χ*^2^ = 2.469, *df* = 1, *p* = 0.116), *O. similis* showed no significant preferences.

### 3.2. Free Choice Test for Oviposition Preference of F. occidentalis

**Figure 2:**
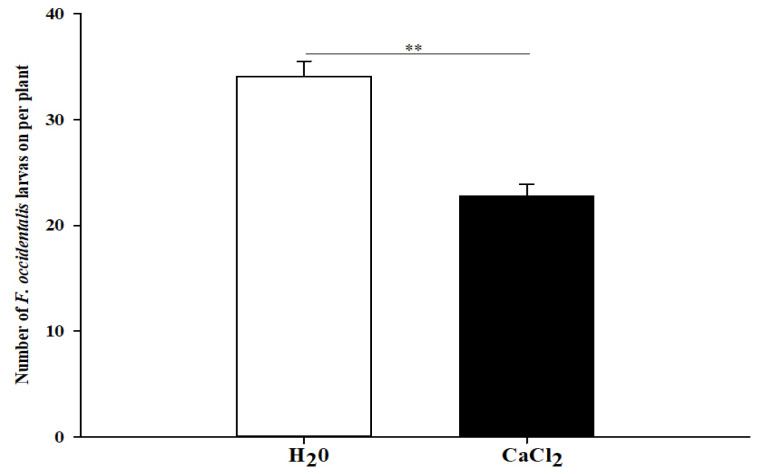
Means (±SE) represent the number of *F. occidentalis* larvae on H_2_O and exogenous calcium-treated kidney bean plants 48 h after oviposition of female *F. occidentalis*. ** indicate high significant difference at 1% level.

The average number of *F. occidentalis* larvae hatched on calcium-treated kidney bean plants was 22.8 thrips, which was significantly lower than that on control plants (34.1 thrips, 33.1% reduction) (Figure 2; *χ*^2^ = 22.441, *df* = 1, N = 10, *p* < 0.01).

### 3.3. Component Analysis of Volatile from Kidney Bean Plant

The types and composition of volatiles differed among the treatment groups. A total of 55 volatile compounds were detected among all treated kidney bean plants. Among them, 35 compounds were detected in the H_2_O treatment group, 40 in each of the CaCl_2_ and H_2_O+WFT treatment groups, and 42 in the CaCl_2_+WFT treatment group (Table 1). 

In plants that received the H_2_O treatment, the component with the highest relative content was 3-hexen-1-ol (28.79% ± 2.02%), which also showed the highest content in the other three treatments. The content of 3-hexen-1-ol in the plants that received the CaCl_2_ treatment was significantly lower than that in the H_2_O and H_2_O+WFT treatments (*F* = 5.850, *p* < 0.05), and there was no significant difference compared with the CaCl_2_+WFT treatment. (E)-2-hexen-1-ol (15.59% ± 0.89%) had the second highest content in the H_2_O+WFT treatment, and the content of (E)-2-hexen-1-ol in the H_2_O+WFT treatment was higher than that in the CaCl_2_ and CaCl_2_+WFT treatments (*F* = 30.360, *p* < 0.05). The content of 1-octen-3-ol in the H_2_O and H_2_O+WFT treatments was not significantly different and was higher than that in the CaCl_2_ and CaCl_2_+WFT treatments (*F* = 25.447, *p* < 0.05). The content of (E)-2-hexenal was higher than 10% in all four treatments, and there was no significant difference among them (*F* = 0.333, *p* > 0.05) (Table 1). The content of β-lonone was the second highest in the CaCl_2_-treated plants and higher than that in the plants treated with the other three treatments (*F* = 72.324, *p* < 0.05). (E,E)-2,4-hexadienal was highest in the CaCl_2_- and CaCl_2_+WFT-treated plants (*F* = 15.408, *p* < 0.05). GC–MS analysis suggested that calcium treatment caused a significant alteration in the proportional composition of (E)-2-hexen-1-ol, 1-octen-3-ol, β-lonone and (E,E)-2,4-hexadienal.

### 3.4. Six-Arm Olfactometer Test

**Figure 3:**
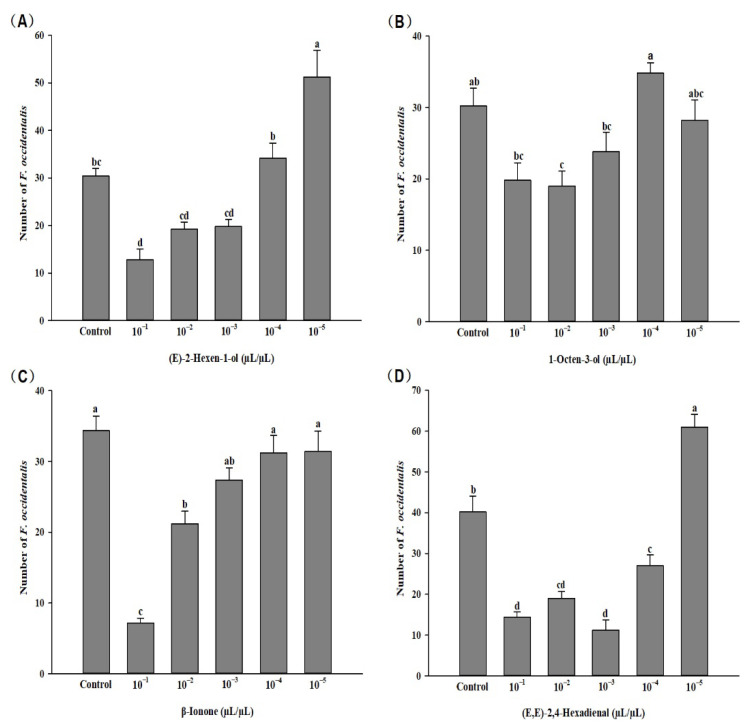
Response of female *F. occidentalis* adults to volatile compounds at different concentrations. Letters on bars indicate significant differences (Tukey’s test, *p* < 0.05).

In the six-arm olfactory test, (E)-2-hexen-1-ol (*F* = 21.268, *p* < 0.01), 1-octen-3-ol (*F* = 6.744, *p* < 0.01), β-lonone (*F* = 24.265, *p* < 0.01), and (E,E)-2,4-hexadienal (*F* = 49.488, *p* < 0.01) showed significant effects on the behavior of *F. occidentalis* at six concentrations. The 10^−1^ μL/μL concentration of (E)-2-hexen-1-ol had the greatest repellent effect on *F. occidentalis*, while the 10^−5^ μL/μL concentration was attractive to thrips. A significant repellent effect on *F. occidentalis* was observed for 1-octen-3-ol at a concentration of 10^−2^ μL/μL. 10^−1^ and 10^−2^ μL/μL β-lonone had significant repellent effect. A significant repellent effect of (E,E)-2,4-hexadienal was observed at concentration of 10^−1^ to 10^−4^ μL/μL, while an attractive effect was observed at the concentration of 10^−5^ μL/μL (Figure 3).

**Figure 4:**
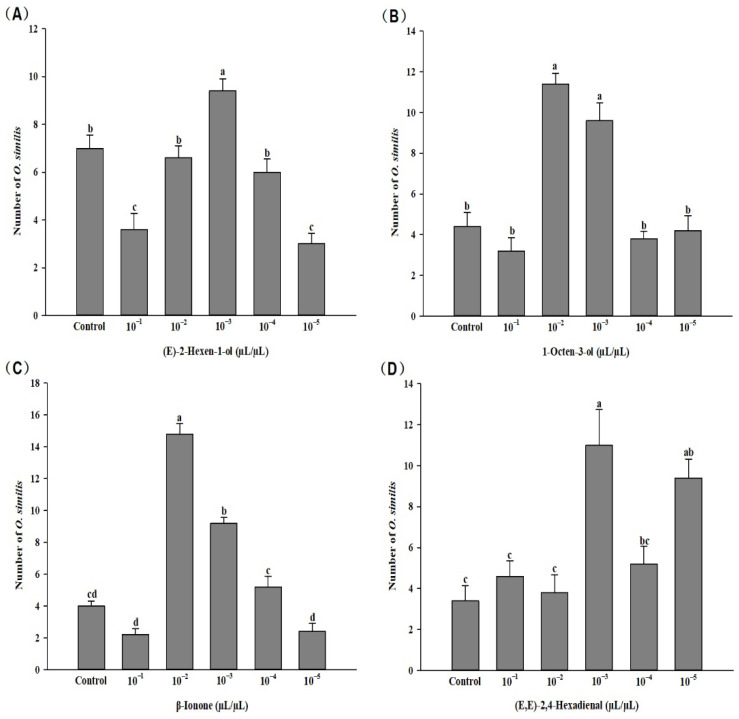
Response of female *O. similis* adults to volatile compounds of different concentrations. Letters on bars indicate significant differences (Tukey’s test, *p* < 0.05).

*O. similis* also showed significant responses to different concentrations of the following four volatile compounds: (E)-2-hexen-1-ol (*F* = 18.643, *p* < 0.01), 1-octen-3-ol (*F* = 27.942, *p* < 0.01), β-lonone (*F* = 94.089, *p* < 0.01), and (E,E)-2,4-hexadienal (*F* = 9.368, *p* < 0.01). Among them, the 10^−3^ μL/μL concentration of (E)-2-hexen-1-ol had significant attractive effect on *O. similis*, while the 10^−1^ and 10^−5^ μL/μL concentrations had repellent effects. The 10^−2^ and 10^−3^ μL/μL concentrations of 1-octen-3-ol had significant attractive effect on *O. similis*. Meanwhile, *O. similis* showed a preference for the 10^−2^ and 10^−3^ μL/μL concentrations of β-lonone. *O. similis* also showed preference for the 10^−3^ and 10^−5^ μL/μL concentrations of (E,E)-2,4-hexadienal. Other concentrations of the compounds did not affect the behavioral responses of *O. similis* (Figure 4).

## 4. Discussion

Calcium has been shown to enhance plant defenses; however, a full understanding of calcium-induced plant defenses requires the determination of the effects of calcium induction on volatile compounds in plants. Because plants, as sessile organisms, cannot avoid contact with herbivores, they produce organic compounds that interfere with herbivore behavior to directly defend themselves [56,57]. For indirect defense, HIPVs are generated to serve as chemical signals for plants to attract predatory or parasitic enemies of herbivores [58,59].

In the Y-tube olfactory experiment, *F. occidentalis* was significantly less attracted to H_2_O+WFT- and CaCl_2_+WFT-treated plants compared with control plants, and CaCl_2_+WFT-treated plants were significantly less attractive than H_2_O+WFT-treated plants, which arose from the altered VOC composition. Previous research has shown that kidney bean plants release HIPVs when infected by thrips, which inhibit the selection of *F. occidentalis* [48]. It has been widely reported that HIPVs affect the behavioral responses of herbivores. For example, after the larvae of *Chilo suppressalis* (Walker) (Lepidoptera: Crambidae) fed on rice plants, the volatiles released from the plants could significantly repel its adults [57]. Cabbage infested with *Spodoptera litura* (Fabricius) (Lepidoptera: Noctuidae) released volatiles to inhibit the selection of *S. litura* larvae [60]. The results of the present study revealed that calcium treatment increased the repellent effect of HIPVs on *F. occidentalis*. Our previous study has also shown that the application of exogenous calcium to kidney bean plants reduces the selectivity of *F. occidentalis* [49]. However, the mechanisms involved remain unclear. Similar findings were found for other exogenous inducers of plant defenses. For example, de Oliveira et al. [61] found that *Rhopalosiphum padi* (Linnaeus) (Hemiptera: Aphididae) preferred constitutive volatiles from control wheat over those emitted by Si-treated wheat plants. Likewise, the application of potassium on soybean led to lower selectivity of soybean aphids compared with control plants [62].

Plant defense responses are not only direct, but they also can likewise work in a roundabout way through the trophic level of herbivore predators [29]. The response of *O. similis* to calcium-treated kidney bean plants was different from that of *F. occidentalis*. Compared with control plants, *O. similis* significantly preferred H_2_O+WFT- and CaCl_2_+WFT-treated kidney bean plants, especially CaCl_2_+WFT-treated plants. CaCl_2_+WFT-treated plants were significantly attractive than H_2_O +WFT-treated plants. Interestingly, *O. similis* showed no preference for volatiles from calcium-treated plants in the absence of thrips infection. This may have been because attracting natural enemies in the absence of a pest population could be counterproductive and compromise biocontrol [63]. Notably, *O. similis* populations were more attracted to plants experiencing thrips infestation if those plants were treated with calcium; this behavior was noted as a result of altered HIPV composition. The attraction of natural enemies to HIPVs is universal [27]. Our previous research indicated that kidney bean plants released HIPVs that could attract *O. similis* when infected by thrips [48]. Our results showed that calcium treatment enhanced the attraction of *O. similis* to HIPVs. Similarly, (Z)-3-hexenol treatment promoted the attraction of the parasitic wasp *Encarsia formosa* (Gahan) (Hymenoptera: Aphelinidae) to its host plant by altering the composition of the HIPVs produced by *B. tabaci* infection [32]. Furthermore, silicon treatment promoted the attraction of *Phytoseiulus persimilis* (Athias-Henriot) (Acari: Phytoseiidae), a natural enemy of *Tetranychus urticae* (Koch) (Acari: Tetranychidae), to host plants by altering the composition of HIPVs produced by *T. urticae* infection [25].

Meanwhile, in the experiment of *F. occidentalis* oviposition selection, it was found that the egg number was significantly reduced in calcium-treated plants (22.8) compared with H_2_O-treated plants (34.1). Such reduced oviposition suggested that calcium-treated plants might lower suitability for *F. occidentalis*. In nature, female insects seek out healthy hosts and more suitable host plants to lay their eggs in order to improve the survival of their offspring, and this process is often carried out by female adults who rely on their sense of smell to recognize special odorous substances emitted by host plants [64,65]. The application of exogenous calcium to kidney bean plants may alter their volatile composition, decreasing the number of thrips on the plants and thus strengthening the continuous control of *F. occidentalis*. In addition, plant secondary metabolites and nutritional status variation may also affect the oviposition preference [66,67], but these factors remain to be investigated in the present study.

GC–MS analysis revealed the number of volatile compounds collected from kidney bean plants of the four different treatment groups. The most abundant volatile compounds were found in plants treated with CaCl_2_+WFT (42 components), followed by those treated with H_2_O+WFT, CaCl_2_, and H_2_O (40, 40, and 35 compounds, respectively). Furthermore, the relative abundance of volatile compounds in the volatile profiles of kidney bean plants differed among the treatment groups. Our study suggested that the CaCl_2_, WFT, and CaCl_2_+WFT treatments increased the number of compound species and altered the relative abundance of plant volatiles. These differences, along with different behavioral responses exhibited by *F. occidentalis* and *O. similis* to different treatments, suggested the high plasticity of the olfactory systems of these insects in detecting suitable host and prey substrates [53]. However, calcium treatment alone does not lead to changes in the behavioral responses of *F. occidentalis* and *O. similis*, indicating that CaCl_2_-mediated plant defense has initiation effects [11,68]. Similar results have been reported in studies using other exogenous inducers, such as silicon and (Z)-3-hexenol, in which treated plants exhibited enhanced defense mechanisms upon infestation with herbivorous *B. tabaci* or *T. urticae* [25,32].

In our study, calcium treatment led to changes in the content of several compounds, among them, the percentages of (E)-2-hexen-1-ol and 1-octen-3-ol were significantly decreased, while the percentages of β-lonone and (E,E)-2,4-hexadienal were significantly increased. In other studies, (E)-2-hexen-1-ol and 1-octen-3-ol have been identified as pest HIPVs, and their increased emissions attract natural enemies of herbivores [69,70]. This finding was contradictory to that of the present study. However, a small number of studies have shown that plants treated with exogenous substances reduced their volatile emissions, causing the enhancement of the attractiveness of natural enemies. For example, the composition of volatiles in silicon-treated rice plants infested with *Cnaphalocrocis medinalis* (Guenee) (Lepidoptera: Pyralidae) was altered, with the content of α-bergamotene, β-sesquiohellandrene, and hexanal 2-ethyl being reduced; however, the attraction of the natural enemies *Trathala flavo-orbitalis* (Cameron) (Hymenoptera: Ichneumonidae) and *Microplitis mediator* (Haliday) (Hymenoptera: Braconidae) was enhanced [59], it has been suggested that this is a function of overall HIPV quality rather than any specific volatile compound [71]. Therefore, we speculated that the altered ratio of compounds in volatile blends was key to the enhanced repellence of herbivorous insects and the attraction of predators. Although the underlying mechanisms and causes of such altered emissions are unclear, we offer possible explanations. The application of exogenous calcium promoted the transduction of signals pathway, including jasmonic acid (JA) and salicylic acid (SA), causing to alter the composition of HIPVs involved in JA or SA [2,5,11,72,73]. Thus, it may attract natural predators and repel herbivorous insects.

When selecting host plants, insects frequently employ certain compounds as cues to locate the plants [64,74]. Therefore, we conducted further behavioral response verification of the volatile components that were significantly altered in calcium treatment kidney bean plants using a six-armed olfactory assay, and found that the different concentrations of (E)-2-hexen-1-ol, 1-octen-3-ol, β-lonone, and (E,E)-2,4-hexadienal had different effects on *F. occidentalis*. (E)-2-hexen-1-ol and (E,E)-2,4-hexadienal compounds showed a repellent effect at higher concentrations and attractant effects at low concentrations. This concentration effect has been similarly observed in previous studies on the repellency of single volatiles; for example, the higher concentrations of 2-phenylethyl acetate acted as a repellent for *F. occidentalis*, but it acted as an attractant at the lower concentrations [75]. Similarly, the higher concentrations of 3-methyl-1-butanol acted as a repellent for *Plodia interpunctella* (Hübner) (Lepidoptera: Pyralidae), but its lower concentrations acted as a lure [76]. This result is attributable to the behavioral responses of herbivores, which depend on the concentration of volatiles [77]. Meanwhile, the concentration effect could also be seen in *O. similis.* Combining the repellent effect on thrips and attraction effect on *O. similis*, 1-octen-3-ol (10^−2^ μL/μL), β-lonone (10^−2^ μL/μL), and (E,E)-2, 4-hexadienal (10^−3^ μL/μL) could be used in biological control against thrips. However, the behavioral responses of insects are not only influenced by individual compounds and concentrations but also by their proportions in a mixture [78]. Thus, it has been suggested that the concentration and ratio of key compounds may influence the attraction of insects [79]. Therefore, the repellent and attraction functions of these volatile compounds should be further evaluated in future studies.

## 5. Conclusions

Our findings revealed that calcium treatment significantly altered the composition of HIPVs in kidney bean plants, affected the oviposition preference of *F. occidentalis*, and attracted the natural enemy of *F. occidentalis*, i.e., *O. similis*. These elements could play roles in the top–down control of *F. occidentalis*. Our results suggested (E)-2-hexen-1-ol, 1-octen-3-ol, β-lonone, and (E,E)-2,4-hexadienal might be potential olfactory cues, but their effects require further evaluation via extensive behavioral bioassay testing using different doses and combinations of these volatile compounds.

## Figures and Tables

**Table 1:** Volatile components of kidney bean plants after four different treatments.

Sn Compounds	Content (%)			
	H_2_O	CaCl_2_	H_2_O+WFT	CaCl_2_+WFT
1 3-hexen-1-ol	28.79 ± 2.02 a	19.24 ± 1.18 b	27.50 ± 2.51 a	23.51 ± 0.99 ab
2 (E)-2-hexen-1-ol	15.59 ± 0.89 a	11.66 ± 1.16 b	17.99 ± 0.54 a	8.01 ± 0.35 c
3 1-octen-3-ol	12.74 ± 0.57 a	8.01 ± 0.35 b	12.31 ± 0.49 a	7.86 ± 0.65 b
4 (E)-2-hexenal	11.40 ± 1.86 a	13.58 ± 1.07 a	13.07 ± 1.19 a	12.63 ± 2.10 a
5 1-hexanol	6.16 ± 0.61 a	4.90 ± 0.51 a	4.95 ± 0.55 a	4.90 ± 0.36 a
6 β-lonone	2.47 ± 0.90 c	15.80 ± 0.82 a	2.69 ± 0.66 c	10.59 ± 0.64 b
7 1-penten-3-ol	1.19 ± 0.27 a	0.36 ± 0.07 b	1.25 ± 0.15 a	0.32 ± 0.04 b
8 Pentadecanal	–	0.48 ± 0.18 b	0.73 ± 0.14 b	2.65 ± 0.63 a
9 β-cyclocitral	0.83 ± 0.03 c	1.73 ± 0.03 a	0.98 ± 0.26 bc	1.63 ± 0.12 ab
10 (Z)-3-hexenyl butanoate	0.98 ± 0.56 a	0.24 ± 0.04 a	0.52 ± 0.06 a	1.31 ± 0.43 a
11 (E,E)-2,4-heptadienal	0.98 ± 0.13 ab	2.07 ± 0.31 a	0.65 ± 0.13 b	1.80 ± 0.34 a
12 3-pentanone	0.47 ± 0.12 a	0.16 ± 0.01 b	0.41 ± 0.01 ab	0.13 ± 0.02 b
13 1-pentanol	0.33 ± 0.19 a	–	0.30 ± 0.12 a	–
14 (E)-2-hexen-1-ol, acetate	0.64 ± 0.07 a	0.30 ± 0.03 b	0.38 ± 0.03 ab	0.44 ± 0.10 ab
15 Nonanal	0.43 ± 0.10 a	0.51 ± 0.01 a	0.43 ± 0.04 a	0.52 ± 0.02 a
16 α-lonone	0.37 ± 0.13 c	1.79 ± 0.18 a	0.83 ± 0.40 ab	1.32 ± 0.15 ab
17 2-ethyl-furan	0.29 ± 0.09 a	0.24 ± 0.03 a	0.35 ± 0.05 a	0.18 ± 0.02 a
18 (E,E)-2,4-hexadienal	0.78 ± 0.52 b	3.95 ± 0.69 a	0.44 ± 0.17 b	3.33 ± 0.22 a
19 3-octanone	0.44 ± 0.06 a	0.26 ± 0.03 a	0.36 ± 0.03 a	0.46 ± 0.12 a
20 2,5-octanedione	0.47 ± 0.25 a	0.62 ± 0.14 a	0.42 ± 0.20 a	0.35 ± 0.15 a
21 2-ethyl-1-hexanol	0.31 ± 0.03 a	0.11 ± 0.01 b	0.24 ± 0.03 a	–
22 Cis-3-hexenyl butyrate	0.34 ± 0.11 b	0.15 ± 0.01 b	0.20 ± 0.01 b	0.67 ± 0.03 a
23 Pentadecane	–	–	0.19 ± 0.04 b	0.73 ± 0.06 a
24 (E,E)-3,5-octadien-2-one	–	–	0.23 ± 0.08 a	0.22 ± 0.02 a
25 Tetradecane	0.13 ± 0.03 c	0.44 ± 0.01 b	0.26 ± 0.08 bc	0.77 ± 0.06 a
26 2-methyl-6-hepten-1-ol	0.22 ± 0.03 a	–	0.20 ± 0.01 a	–
27 β-cyclohomocitral	0.16 ± 0.01 b	0.51 ± 0.03 a	0.21 ± 0.05 b	0.36 ± 0.04 a
28 Benzeneacetaldehyde	0.13 ± 0.02 b	0.20 ± 0.01 ab	0.13 ± 0.02 b	0.27 ± 0.02 a
29 Hexadecane	–	–	–	0.10 ± 0.06
30 Cis-3-hexenyl Cis-3-hexenoate	–	–	0.16 ± 0.03 b	0.58 ± 0.10 a
31 3-ethylbenzaldehyde	0.12 ± 0.04 b	0.62 ± 0.04 a	0.13 ± 0.03 b	0.47 ± 0.07 a
32 (4H)-benzofuranone, 5,6,7,7a-tetrahydro-4,4,7a-trimethyl-,(R)-	–	0.66 ± 0.06 a	–	0.42 ± 0.05 b
33 2,2,6-trimethylcyclohexanone	0.10 ± 0.02 a	–	0.10 ± 0.02 a	–
34 Benzaldehyde	0.15 ± 0.02 b	0.33 ± 0.02 a	–	0.31 ± 0.02 a
35 2,6-di-tert-butylbenzoquinone	0.12 ± 0.04 b	0.28 ± 0.02 a	–	0.27 ± 0.02 a
36 (E,E)-2,6-nonadienal	0.13 ± 0.06 a	0.15 ± 0.03 a	0.10 ± 0.04 a	0.18 ± 0.03 a
37 Tridecane	–	0.13 ± 0.02 ab	–	0.21 ± 0.03 a
38 Decanal	–	0.26 ± 0.04 a	–	0.19 ± 0.01 a
39 (E)-geranylacetone	–	0.24 ± 0.03 a	–	0.21 ± 0.04 b
40 Butanoic acid, hexyl ester	–	–	–	0.13 ± 0.02 a
41 Perhydrofarnesyl acetone	–	0.13 ± 0.02	–	–
42 Hexadecanoic acid, methyl ester	–	0.20 ± 0.04 a	–	0.11 ± 0.02 a
43 (Z)-3-hexen-1-ol, acetate	4.37 ± 1.70 a	0.68 ± 0.03 a	1.22 ± 0.75 a	2.64 ± 0.72 a
44 Lauryl acetate	–	–	–	0.24 ± 0.12
45 (E)-2-penten-1-ol	–	–	0.24 ± 0.03	–
46 (Z)-2-penten-1-ol	–	–	0.57 ± 0.17	–
47 Linalool	–	–	0.11 ± 0.05	–
48 3-methyl-butanal	0.19 ± 0.03 a	–	0.21 ± 0.01a	–
49 1-nonanol	0.14 ± 0.01 a	0.20 ± 0.03 a	0.21 ± 0.04 a	–
50 Hexanal	0.14 ± 0.01 a	0.20 ± 0.03 a	0.21 ± 0.04 a	–
51 1-heptanol	–	–	0.13 ± 0.00	–
52 3-hexenal	–	0.34 ± 0.01 a	–	0.35 ± 0.06 a
53 (E)-3-hexen-1-ol	–	–	–	0.37 ± 0.02
54 1-tetradecene	–	0.38 ± 0.01	–	–
55 (Z)-2-penten-1-ol	0.74 ± 0.21 a	0.46 ± 0.02 a	–	0.34 ± 0.04 a

The data in the table are presented as mean ± standard error, and lower-case letters indicate significant differences among different concentrations and compounds (Tukey’s test, *p* < 0.05).

## Data Availability

Data is available on request.
